# Measuring heritable contributions to Alzheimer’s disease: polygenic risk score analysis with twins

**DOI:** 10.1093/braincomms/fcab308

**Published:** 2022-01-04

**Authors:** Ida K. Karlsson, Valentina Escott-Price, Margaret Gatz, John Hardy, Nancy L. Pedersen, Maryam Shoai, Chandra A. Reynolds

**Affiliations:** Department of Medical Epidemiology and Biostatistics, Karolinska Institutet, Stockholm, Sweden; Aging Research Network—Jönköping (ARN-J), School of Health and 6 Welfare, Jönköping University, Jönköping, Sweden; UK Dementia Research Institute at Cardiff, Institute of Psychological Medicine and Clinical Neurosciences, Cardiff University, Cardiff, UK; Department of Medical Epidemiology and Biostatistics, Karolinska Institutet, Stockholm, Sweden; Center for Economic and Social Research, University of Southern California, Los Angeles, CA, USA; Department of Neurodegenerative Disease, UCL Queen Square Institute of Neurology, Queen Square, London WC1N 3BG, UK; UK Dementia Research Institute at UCL and Department of Neurodegenerative Disease, UCL Institute of Neurology, University College London, London, UK; Reta Lila Weston Institute, UCL Queen Square Institute of Neurology, 1 Wakefield Street, London WC1N 1PJ, UK; UCL Movement Disorders Centre, University College London, London, UK; Institute for Advanced Study, The Hong Kong University of Science and Technology, Hong Kong SAR, China; Department of Medical Epidemiology and Biostatistics, Karolinska Institutet, Stockholm, Sweden; Department of Psychology, University of Southern California, Los Angeles, CA, USA; Department of Neurodegenerative Disease, UCL Queen Square Institute of Neurology, Queen Square, London WC1N 3BG, UK; UK Dementia Research Institute at UCL and Department of Neurodegenerative Disease, UCL Institute of Neurology, University College London, London, UK; Department of Psychology, University of California—Riverside, Riverside, CA, USA

**Keywords:** polygenic risk scores (PRSs), *APOE*, heritability, twins, Alzheimer’s disease

## Abstract

The heritability of Alzheimer’s disease estimated from twin studies is greater than the heritability derived from genome-based studies, for reasons that remain unclear. We apply both approaches to the same twin sample, considering both Alzheimer’s disease polygenic risk scores and heritability from twin models, to provide insight into the role of measured genetic variants and to quantify uncaptured genetic risk. A population-based heritability and polygenic association study of Alzheimer’s disease was conducted between 1986 and 2016 and is the first study to incorporate polygenic risk scores into biometrical twin models of Alzheimer’s disease. The sample included 1586 twins drawn from the Swedish Twin Registry which were nested within 1137 twin pairs (449 complete pairs and 688 incomplete pairs) with clinically based diagnoses and registry follow-up (*M*_age_ = 85.28, SD* *= 7.02; 44% male; 431 cases and 1155 controls). We report contributions of polygenic risk scores at *P *< 1 × 10^−5^, considering a full polygenic risk score (PRS), PRS without the *APOE* region (PRS.no.APOE) and PRS.no.APOE plus directly measured *APOE* alleles. Biometric twin models estimated the contribution of environmental influences and measured (PRS) and unmeasured genes to Alzheimer’s disease risk. The full PRS and PRS.no.APOE contributed 10.1 and 2.4% to Alzheimer’s disease risk, respectively. When *APOE* ɛ4 alleles were added to the model with the PRS.no.APOE, the total contribution was 11.4% to Alzheimer’s disease risk, where *APOE* ɛ4 explained 9.3% and PRS.no.APOE dropped from 2.4 to 2.1%. The total genetic contribution to Alzheimer’s disease risk, measured and unmeasured, was 71% while environmental influences unique to each twin accounted for 29% of the risk. The *APOE* region accounts for much of the measurable genetic contribution to Alzheimer’s disease, with a smaller contribution from other measured polygenic influences. Importantly, substantial background genetic influences remain to be understood.

## Teaser

Karlsson *et al*. report that measured polygenic scores from genome-based studies, including an outsized role for *APOE*, explain only a fraction of the heritability indicated by twin models of Alzheimer’s disease, leaving most genetic risk for Alzheimer’s disease unexplained. Sensitive designs are needed to capture all the genetic influences.

## Graphical Abstract

**Figure:**
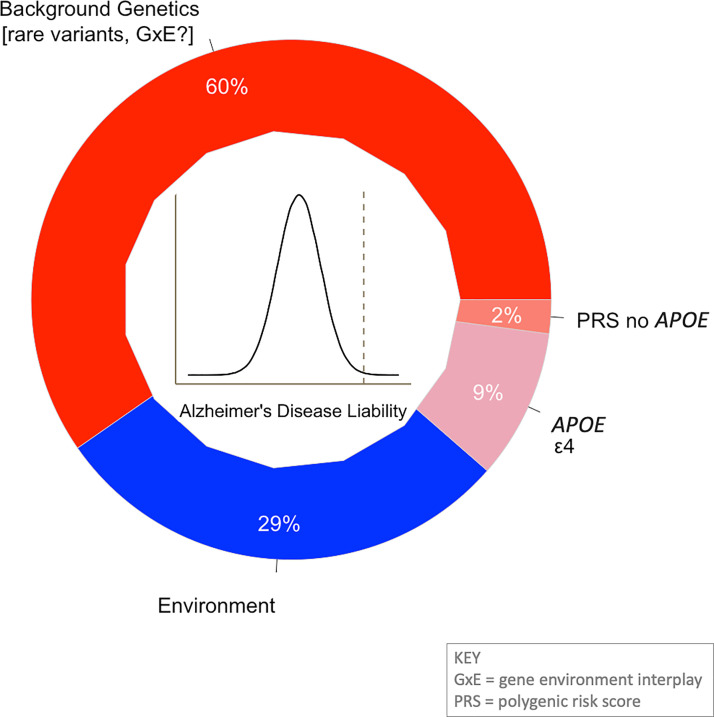

## Introduction

Alzheimer’s disease is multifactorial with contributions of genetic and environmental influences. Twin studies leveraging the relative similarity of Alzheimer’s disease risk among identical or monozygotic (MZ) versus fraternal or dizygotic (DZ) twin pairs suggests an overall heritability of 0.58, with a maximum heritability of 0.79 if shared environmental influences are discounted.^1^ Thus, 58–79% of the liability to late-onset Alzheimer’s disease is heritable. By comparison, measured loci contributing to late-onset Alzheimer’s disease risk may capture up to 50% of the heritability.^2^ However, the comparability of estimates remains unclear as the estimation of polygenic contribution varies across study designs. We sought to provide insight into the role of *APOE*, which codes for apolipoprotein E, the major cholesterol transporter in the brain, and other measured genetic variants using polygenic risk scores (PRSs), as well as quantify uncaptured genetic risk in Alzheimer’s disease, within the same sample of twins.

The application of the PRS approach, a weighted sum of single nucleotide polymorphism (SNP) variants based on the effect sizes from genome-wide association study (GWAS), leads to enhanced accuracy in the prediction of Alzheimer’s disease risk. For example, in case–control samples from the GERARD consortia, the best prediction accuracy using area under the curve (AUC) was 0.78 (0.77–0.80) based on a logistic regression model with measured apolipoprotein E (*APOE*) genotypes, a PRS comprising 20 SNPs from the Lambert *et al.*^3^ GWAS meta-analysis, sex and age.^4^  *APOE* ɛ4 alone achieves an AUC of about 0.68^5^; however, when *APOE* ɛ4 carriers are excluded, the prediction accuracy of the PRS achieves an AUC of 0.65.^5^ That is, PRS prediction of risk is substantial even for those who do not carry the ɛ4 allele. Moreover, AUC model-based inferred heritability from maximum prediction models^6^ suggests that in neuropathologically confirmed cases and controls, heritability estimates can be inferred to lie between 27 and 55%^7^ based on common genome-wide SNPs contributing to liability and accounting for age-related increases in prevalence. This range is in line with other estimates of SNP-based heritability of 24–53%, with *APOE* ɛ4 accounting for approximately one-quarter of the genetic contributions to liability.^8,9^ Apart from *APOE*, other genes identified in recent GWAS are involved in amyloid precursor protein (APP) metabolism/β-amyloid (Aβ) formation and regulation of APP catabolic process, τ-protein binding, lipid metabolism and immune response.^10,11^

How much heritable variation a PRS captures for Alzheimer’s disease risk may be related to its genetic architecture. Recent work suggests that Alzheimer’s disease may be oligogenic, or influenced by a limited set of common genetic variants compared with other complex traits.^12^ However, the age distribution among Alzheimer’s disease cases versus controls, and thus differences in the prevalence of *APOE* ɛ2 versus ɛ4 allele frequencies can impact PRS prediction.^13^ In addition to Alzheimer’s disease risk, *APOE* is associated with longevity where the allele frequencies for ɛ2 become more prevalent in older samples and ɛ4 alleles become less prevalent, at least in samples of European and Asian ancestries.^14–17^ Moreover, the methods used to construct PRSs for Alzheimer’s disease can impact the composition of genetic variants included and hence prediction. A PRS constructed from a clumping and *P*-value threshold approach PRS(C + T) and related methods outperform or are comparable with other approaches (e.g. LDPRED and SBayesR).^13^ The best prediction was observed in a model combining directly measured *APOE* with the PRS excluding the *APOE* region at a threshold of *P *≤ 0.10, whereas the prediction accuracy was attenuated at more relaxed thresholds despite increases in variants.^13^ Altogether, recent findings suggest that Alzheimer’s disease is polygenic and the age-related nature of the risk is essential to consider.^13^

The gap between heritability estimates from genome-based and twin-based studies is notable, although the upper range of genome or SNP-based heritability is at the cusp of heritability estimates observed in twin studies. That said, genome-based and twin-based estimates capture discrete components. While twin analyses typically model additive genetic effects, these estimates capture both additive and non-additive genetic variance shared among twins as well as gene–environment interplay, and contributions from both rare and common variants (and often is referred to as ‘broad-sense heritability’),^18^ whereas genome-based methods capture additive variance attributable to informative common genetic variants on genotyping arrays (known as ‘narrow-sense heritability’).^8^ In the current study, we implement two methods within the same twin samples and evaluate how Alzheimer’s disease PRS contributions to heritability vary and what Alzheimer’s disease PRS contributes beyond *APOE*.

## Materials and methods

### Participants

All participants were drawn from the Swedish Twin Registry (STR).^19^ The primary analysis sample included twins from four STR-based sub-studies: The Study of Dementia in Swedish Twins (HARMONY),^20^ the Swedish Adoption Twin Study of Aging (SATSA),^21^ Aging in Women and Men (GENDER)^22^ and Origins of Variance in the Oldest Old: Octogenarian Twins (OCTO-Twin),^23^ where informed consent was obtained from participants. Dementia was assessed using equivalent protocols that permits the combining of these data.^24,25^ SATSA, begun in 1984, followed 859 individuals aged 50 years and older from same-sex pairs across three decades with 10 in-person testing assessments commencing in 1986^21^; the current analysis sample included 522 SATSA participants. OCTO-Twin, initiated in 1991, followed 351 same-sex twin pairs aged 80 years and older across 8 years with five biennial visits^23^; the current analysis sample included 66 OCTO-Twin participants. GENDER, initiated in 1995 includes three in-person follow-ups of 498 opposite-sex twin pairs aged 70 years and older^22^; the current analysis sample included 326 GENDER participants. HARMONY, commencing in 1998, screened 13 939 individuals from all STR individuals aged 65 years and older.^20^ Those who evidenced possible cognitive dysfunction were referred for a complete clinical work-up as well as their co-twin, plus a control sample, with a total clinical sample of 1557. A longitudinal follow-up after 2 years was done of those in the clinical work-up samples who showed possible dysfunction but did not meet the criteria for dementia. The current analysis sample included 666 HARMONY participants. Clinically based dementia and Alzheimer’s disease diagnoses were available from the in-person evaluations^1^ beginning in 1986 with additional follow-up through population-based registries up through 2016. Diagnoses available via registry sources are reliable.^26^

For individuals diagnosed with dementia, age at dementia diagnosis was used as the last follow-up. For controls, age at last follow-up was based on the age as on 31 December 2016 or death, whichever occurred first for those with register information as described below. The age at last follow-up, death or dementia onset was *M*_lastage_ = 85.28, SD = 7.02 years with 44% of the sample being male. Age distributions across cases and controls are similar although controls are on an average 2.32 years younger than the cases (see Supplementary Table 1). Age distributions within the sub-studies are generally similar among cases and controls overall, with average age differences between controls and cases ranging from −5.11 to 0.45 years, with the largest difference for SATSA.

Twins were selected for analyses where one or both members of the pair had information about a diagnosis consistent with Alzheimer’s disease or mixed Alzheimer’s disease and *APOE* genotyping. Exclusions included early-onset Alzheimer’s disease cases (aged <60 years, *n = *3) and individuals with other forms of dementia (*n = *382). Controls were excluded if they died before the age of 70 years (*n = *38) or if they had possible cognitive impairment but did not meet the criteria for dementia (*n = *110). Additional exclusionary criteria included no genome-wide genotyping (*n = *76) or undetermined zygosity (*n = *7). After these exclusions, a total of 1586 twins were available for the analytic samples (431 Alzheimer’s disease or mixed Alzheimer’s disease cases, 1155 controls). The 1586 twins were nested within 1137 twin pairs, with 898 individuals represented among 449 complete pairs and 688 individuals represented from 688 incomplete pairs.

### Measures

#### Alzheimer’s disease assessment

A two-stage procedure identified dementia cases. First, cognitive screening by telephone was performed across the entire STR population by HARMONY or where twins missed a longitudinal assessment (SATSA, OCTO-Twin and GENDER), or where longitudinal performance declined markedly (e.g. mental status performance via a Mini-Mental Status Exam (MMSE)^27^ score <25 or a longitudinal drop by three points; low cognitive performance on verbal or spatial tasks in the bottom 10th percentile or dropping the equivalent of 1 SD from the prior assessment). Second, poor performance on the screening led to referral for in-person dementia diagnostic work-up for those twins, along with their cotwins.^1^ All studies also worked up samples of twin pairs who did not perform poorly on the cognitive screening. For individuals lost to follow-up due to the end of the parent study, or if a twin skipped an assessment wave, administrative sources were consulted, including the Swedish National Patient Register, the Cause of Death Register and the Prescribed Drug Register. The present study updated dementia status through 31 December 2016, using International Classification of Disease codes for Alzheimer’s disease and other dementias or Anatomical Therapeutic Chemical codes for Alzheimer’s disease medication (used as a proxy for an Alzheimer’s disease diagnosis).^28^

#### Genotyping

Direct *APOE* genotyping for two markers (rs7412 and rs429358) was available for all participants included in the analysis as described elsewhere.^29^ The distribution of *APOE* ɛ2/ɛ3/ɛ4 alleles in this analysis sample was 9.4/74.2/16.4% (taking all DZ twins and selecting one individual from each MZ pair). Genome-wide data were available from the Illumina PsychArray (*N* = 1451) or the Human OmniExpress array (*N* = 135) and imputed to 1000 Genomes Project phase1 version3.^30^ Initial exclusions of SNPs included those with a minor allele frequency of 0, >2% missing calls and those out of Hardy–Weinberg equilibrium (*P* < 1 × 10^−6^). Ancestral outliers (based on principal components) and individuals with >1% missing genotypes were excluded. PRSs were created in Plink 1.9^31^ using summary statistics from the 2019 Alzheimer’s disease genetic meta-analysis.^10^ All non-ambiguous SNPs in the summary statistics were selected for PRS generation if they were also present in the study sample data with a minor allele frequency of 1% or higher and info score >0.8 (indicating good imputation quality) on both genotyping arrays. Using Plink 1.9,^31^ independent genetic variants were obtained through linkage disequilibrium (LD) clumping, setting the LD parameter *r*^2^ to 0.01. PRSs were then computed by summing up the number of risk alleles at each SNP, weighted by the effect size from the GWAS summary statistics.^31^ Eight different PRSs were computed based on significance level in the GWAS, at *P* ≤ 1, *P* ≤ 0.5, *P* ≤ 0.05, *P* ≤ 0.01, *P* ≤ 1 × 10^−3^, *P* ≤ 1 × 10^−4^, *P* ≤ 1 × 10^−5^ and *P* ≤ 5 × 10^−8^, with and without the *APOE* region. For 183 of the MZ twin pairs, only one twin was genotyped and the co-twin’s PRS imputed by taking the genotyped twin’s PRS.

### Analysis

Regression analyses included both complete and incomplete pairs (*N* = 1586 individuals from 1137 twin pairs), whereas biometric models included complete pairs (*N* = 898 individuals, 449 pairs). PRSs were adjusted for the first four ancestry principal components and standardized within the SNP array.

PRS effects in a regression context were tested using the R package *mixor*^32^ (v.1.04) using a probit model as follows:

(1) $$\mathrm{probit}(AD_{ij})=b_{0}+b_{1}\mathrm{MZ}+b_{2}\mathrm{Sex}+b_{3}\mathrm{LastAge}+b_{4}\mathrm{LastAg}e^{2}+b_{5}\mathrm{Array}+b_{6}\mathrm{zPRS}$$

where AD reflects Alzheimer’s disease risk for the *i*th individual in the *j*th pair as predicted by an MZ twin type, Sex, LastAge (centered on 80 years, divided by 10), Array (Omni or Psych) and zPRS the residualized and standardized PRS scores. Random effects for MZ and DZ pairs were estimated at the pair level to account for sibling dependencies. Fit comparisons between a baseline model with covariates and adding the PRS or *APOE* alleles were made comparing deviances distributed as chi-square (Δχ^2^) with d.f. equal to the number of predictors added to the model. The probit model was prioritized as it underlies the biometrical model described below. However, a model assuming a logit link produced comparable estimates and is presented in Supplementary material for comparison with previously published work.

**Figure 1:**
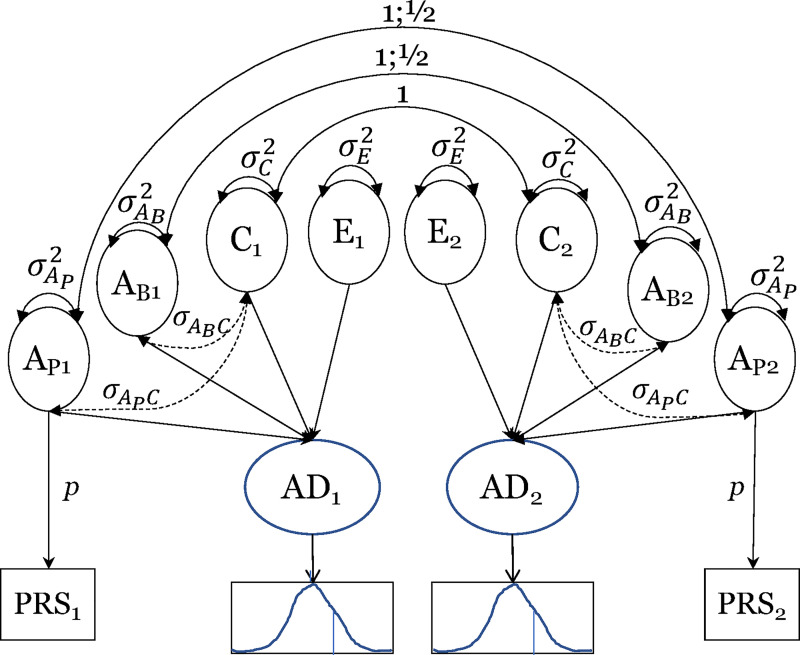
**Biometrical ACE model with Alzheimer’s disease PRS.** AD, Alzheimer’s disease liability; PRS, polygenic risk score. *A*_P_, additive genetic influences due to the PRS which are correlated at 1.0 among MZ twin pair members and 1/2 for DZ twins pair members; *A*_B_, background additive genetic influences which are correlated at 1 among MZ and 1/2 for DZ twin pairs; *C*, common environmental influences that are perfectly correlated among both MZ and DZ pairs; *E*, non-shared environmental influences. Subscripts of 1 refer to Twin 1 and subscripts of 2 refer to Twin 2. Total *A* = *A*_P_ + *A*_B_ + 2cov*AC*, where cov*AC* is the total covariance of *A* and *C*.

PRS contributions in the context of a biometric model were tested using the R package *OpenMx*^33^ (v. 2.18.1), assuming a latent-liability probit model with maximum-likelihood estimation. We fitted an extended ACE biometric twin model^34^ (see Fig. 1), decomposing underlying liability to Alzheimer’s disease into total additive genetic (*A*) influences, common (*C*) and non-shared or person-specific environmental (*E*) influences, and covariance between *A* and *C* (cov*AC*). Notably, *E* also includes any measurement error and stochastic factors. Additive genetic influences include the unmeasured background genetic (*A*_B_) component and a latent polygenic risk score (*A*_P_) that was perfectly defined by the measured PRS and its observed variance scaled by the parameter *p* (i.e., σ^2^_PRS_ = *p*^2^ x σ^2^_*A*ᴩ_). An identifying constraint included no covariance between *A*_B_ and *A*_P_ (σ_*A*ᴩ,*A*ʙ_ = 0). The sum of variance components was constrained such that

(2) $$1=\sigma_{A_{B}}^{2}+\sigma_{A_{P}}^{2}+\sigma_{C}^{2}\;+2\sigma_{AC}+\sigma_{E}^{2}$$

Hence, σ^2^_*A*__ᴩ_ represents the proportion of variance in Alzheimer’s disease liability explained by the measured PRS and σ^2^_*A*ᴩ_ + σ^2^_*A*ʙ_ represents the proportion of variance due to all genetic influences. In addition, the total covariance between *A* and *C* (cov*AC*) was constrained as:

(3) $$\sigma_{AC}=\;\sigma_{A_{P}C}+\sigma_{A_{B}C}\;$$

Hence, the expected correlations among MZ twins who share 100% of their genes while DZ twins on average share 50% of their segregating alleles were:

(4) $$r_{\mathrm{MZ}}=\sigma_{A_{B}}^{2}+\sigma_{A_{P}\;}^{2}+\sigma_{C}^{2}\;+2\sigma_{AC}$$

(5) $$r_{\mathrm{DZ}}=\frac{1}{2}\sigma_{A_{B}}^{2}+\frac{1}{2}\sigma_{A_{P}\;}^{2}+\sigma_{C}^{2}\;+2\sigma_{AC}$$

The models freely estimated variance components without boundary constraints to allow for unbiased fit statistics and correct Type I error rates.^35^ We fixed the Alzheimer’s disease liability threshold to 0 and estimated its mean for ease in analysis given that the mean estimation was already specified for the PRSs, and is a statistically equivalent approach to estimating the threshold and fixing the mean to 0.^36^ 95% confidence intervals were estimated.

### Data availability

Raw data were generated at the Department of Medical Epidemiology and Biostatistics, Karolinska Institutet, Stockholm, Sweden. The derived data supporting the findings of this study are available from the corresponding author on request.

## Results

In comparing the IGAP2 summary statistics^10,11^ with GWAS of our current samples, the *β* coefficients suggested similar effect sizes for those included in the PRS at *P *< 1 × 10^−5^ (*r*_GWAS _= 0.54, *P* < 6.1 × 10^−8^; *n*_SNPs _= 89) and *P *< 1 × 10^−4^ (*r*_GWAS _= 0.36, *P* < 2.1 × 10^−8^; *n*_SNPs _= 233). The best regression predictions were observed at *P *< 1 × 10^−5^ and *P *< 1 × 10^−4^ thresholds (Nagelkerke *R*^2 ^= 0.062 for both) and improved over *P *< 5 × 10^−8^ (Nagelkerke *R*^2 ^= 0.058), whereas predictions fell off at *P *< 1 × 10^−3^ (Nagelkerke *R*^2 ^= 0.053) (see Supplemental Table 2 for all thresholds). The comparable predictions based on the PRS without the *APOE* region were Nagelkerke *R*^2^ values of 0.011 and 0.012 at the *P *< 1 × 10^−5^ and *P* < 1 × 10^−4^ thresholds, respectively. We present findings for the *P *< 1 × 10^−5^ threshold (based on the *r*_GWAS_ and comparable Nagelkerke *R*^2^), evaluating a full PRS, a PRS without the *APOE* region (PRS.no.APOE) and the latter with directly measured *APOE* alleles (PRS.no.APOE + ɛ2 + ɛ4 alleles).

### Probit regression models

Entering the full PRS at *P *< 1 × 10^−5^ to the baseline model with covariates led to a significant increase in fit [Δχ^2^_(d.f. = 1) _= 65.82, *P *< 4.93 × 10^−16^; Nagelkerke *R*^2 ^= 0.062] (Table 1). Entering PRS.no.APOE also led to a significant increase in fit [Δχ^2^_(d.f. = 1) _= 11.27, *P* < 7.86 × 10^−4^; Nagelkerke *R*^2 ^= 0.011] (Table 1). When adding directly genotyped *APOE* ɛ2 alleles and ɛ4 alleles (PRS.no.APOE + ɛ2 + ɛ4 alleles), the resulting gain in prediction was evident [Δχ^2^_(d.f. = 2) _= 81.29, *P *< 2.23 × 10^−18^] with a Nagelkerke *R*^2^ of 0.076, driven by *APOE* ɛ4 (*P *= 2.05 × 10^−12^) and with a non-significant reduction in risk by the number of ɛ2 alleles (*P *= 1.38 × 10^−1^) (Table 1). The AUC values across all models were high ranging from 0.97 to 0.98, suggesting that background characteristics perform well in distinguishing cases from non-cases. Logistic regression models produced similar results (see Supplementary Table 3). Sensitivity analyses using only complete twin pairs produced consistent results as the full sample analysis (see Supplementary Table 4). Finally, analyses adding in adjustment for sub-study resulted in slight differences: the Nagelkerke *R*^2^ dropped from 0.062 to 0.055 = 0.007 for PRS with *APOE* and from 0.011 to 0.009 for PRS.no.APOE (see Supplementary Table 5). Overall, the best genetic prediction was observed for directly measured *APOE* ɛ2 and ɛ4 plus PRS.no.APOE (Table 1).

**Table 1:** Probit regression analyses (*N* = 1586): Alzheimer’s disease PRS at *P *< 1 × 10^−5^

Parameters	Baseline			PRS *P *< 1 × 10^−5^			PRS.no.APOE *P *< 1 × 10^−5^			PRS.no.APOE + *APOE* alleles		
	*B*	SE	*P*(>|*z*|)	*B*	SE	*P*(>|*z*|)	*B*	SE	*P*(>|*z*|)	*B*	SE	*P*(>|*z*|)
Intercept	−1.56	0.20	4.89E−15	−1.67	0.21	8.88E−16	−1.58	0.20	2.89E−15	−1.82	0.22	<2.20E−16
MZ	0.04	0.16	7.92E−1	0.06	0.15	6.79E−1	0.08	0.16	6.15E−1	0.07	0.15	6.08E−1
Sex (0 = M, 1 = F)	0.44	0.09	3.83E−6	0.44	0.10	4.15E−6	0.44	0.09	3.02E−6	0.41	0.09	1.40E−5
LastAge	0.97	0.12	8.88E−15	0.97	0.13	2.75E−14	0.95	0.13	4.31E−14	0.96	0.13	6.51E−14
LastAge^2^	−0.55	0.09	5.12E−10	−0.50	0.09	1.29E−8	−0.53	0.09	1.78E−9	−0.47	0.09	4.27E−8
Array	0.40	0.16	1.55E−2	0.44	0.17	9.83E−3	0.40	0.17	1.69E−2	0.38	0.17	2.88E−2
PRS	—	—	—	0.38	0.06	7.53E−12	0.16	0.05	1.41E−3	0.16	0.05	1.06E−3
*APOE* ɛ2 alleles	—	—	—	—	—	—	—	—	—	−0.18	0.12	1.38E−1
*APOE* ɛ4 alleles	—	—	—	—	—	—	—	—	—	0.75	0.11	2.05E−12
Random·MZ	2.23	0.76	3.37E−3	1.77	0.62	4.49E−3	2.06	0.73	4.53E−3	1.61	0.58	5.60E−3
Random·DZ	0.37	0.23	1.04E−1	0.39	0.24	1.02E−1	0.39	0.24	1.02E−1	0.36	0.24	1.40E−1
**Fit statistics**												
Deviance	1703.37			1637.55			1692.10			1610.81		
AIC	−859.69			−827.78			−855.05			−816.40		
SBC	−879.83			−850.44			−877.71			−844.10		
ICC·MZ	0.691			0.638			0.673			0.616		
ICC·DZ	0.269			0.282			0.280			0.263		
AUC	0.976			0.972			0.977			0.970		
* R* ^2^ Nagelkerke	0.084			0.062			0.011			0.076		

Regression analyses estimating varying intraclass correlations (ICCs) with clustered twin data were adapted from code in Archer *et al.*^32^ using *mixor.* MZ, monozygotic twin; DZ, dizygotic twin; LastAge, age at last follow-up, death or dementia onset, centred on age 80 years and divided by 10; Array, Human OmniExpress* *=* *0, Illumina PsychArray* *=* *1; PRS, polygenic risk score at *P *< 1 × 10^−5^ residualized for four PCs and standardized within array type; PRS.no.APOE, PRS without *APOE* region; Random, random effect; Deviance, –2ln(Likelihood); AIC, Akaike Information Criteria; SBC, Schwarz Bayesian Criterion; ICCs measured as Random·MZ/(1* *+* *Random·MZ) and Random·DZ/(1* *+* *Random·DZ)^32^; AUC, area under the curve.

**Figure 2:**
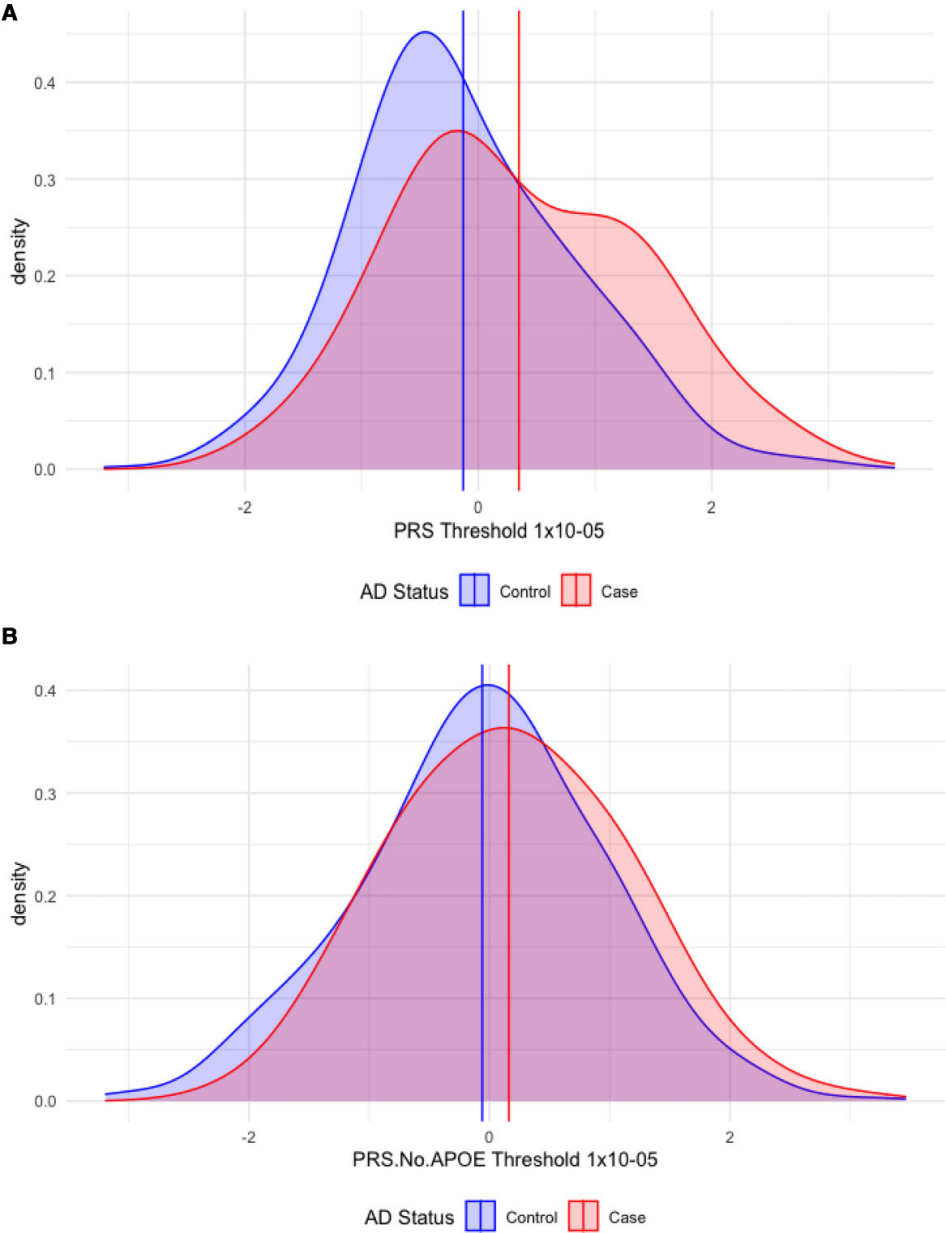
**Density plots of Alzheimer’s disease PRSs at the *P *< 1 × 10^−5^ threshold in cases and controls.** (**A**) PRS. (**B**) PRS.no.APOE (PRS without *APOE* region). PRS, polygenic risk score. Vertical lines indicate the mean PRS values for Alzheimer’s disease (AD) cases (red line) and controls (blue line). PRSs are based on independent genetic variants reaching a significance threshold of *P *< 1 × 10^−5^ in the GWAS.

The standardized PRS distribution at *P *< 1 × 10^−5^, by Alzheimer’s disease status, is shown in Fig. 2A, adjusted for the first four ancestry PCs and array type. The mean PRS for controls was −0.13 (SD = 0.95) versus cases at 0.35 (SD = 1.06), an effect size difference of *z* = 0.48. The standardized PRS distribution for PRS.no.APOE at *P *< 1 × 10^−5^, by Alzheimer’s disease status, is shown in Fig. 2B, adjusted for the first four ancestry PCs and array type. The mean PRS.no.APOE for controls was −0.06 (SD* *= 1.00) versus cases at 0.16 (SD = 0.97), an effect size difference of *z *= 0.22. Hence, the offset in the PRS distributions between cases and controls is over 2-fold for the full PRS containing the *APOE* region compared with the distribution of PRS.no.APOE.

### Biometric twin models

A simple baseline ACE model fitted to complete twin pairs (190 MZ and 259 DZ) suggested a significant additive genetic contribution (*A*), a non-significant common environmental variance (*C*), and a significant non-shared or person-specific environmental variance (*E*) (see Table 2, Full Model 0). A reduced model dropping common environmental variance (*C*) [Δ*χ*^2^_(d.f. = 1) _= 0.81, *P *= 3.69 × 10^−1^] was the best-fitting and suggested that 70.7% of the liability for Alzheimer’s disease was attributable to additive genetic influences (σ^2^_*A*_ = 0.707, CI_95 _= 0.542, 0.832) and the remainder attributable to non-shared environment (Table 2, Reduced Model 0). Model fit comparisons between the full baseline ACE and all sub-models testing individual variance components are shown in Supplementary Table 6.

**Table 2:** Biometric twin model results: Alzheimer’s disease PRS at *P *< 1 × 10^−5^

Model	VC	Full model				Reduced model			
		Est	SE	LL	UL	Est	SE	LL	UL
0. ACE	*A*	0.960	0.294	0.391	1.546	0.707	0.074	0.542	0.832
	*C*	−0.232	0.265	−0.774	0.258	—	—	—	—
	*E*	0.272	0.073	0.153	0.440	0.293	0.074	0.168	0.458
	−2LL	888.71				889.52			
	AIC	902.71				901.52			
	BIC	931.46				926.16			
1. PRS	*A*_P_	0.136	0.040	0.058	0.342	0.101	0.029	0.051	0.164
	*A*_B_	0.882	0.083	0.343	1.410	0.614	0.075	0.452	0.744
	*C*	0.003	4.26E−4	−9.31E−7	0.668	—	—	—	—
	*E*	0.263	0.069	0.149	0.425	0.285	0.072	0.164	0.446
	cov*AC*	−0.142	0.008	−0.618	0.098	—	—	—	—
	−2LL	2746.32				2747.52			
	AIC	2768.32				2765.52			
	BIC	2813.49				2802.48			
2. PRS.no.APOE	*A*_P_	0.030	0.020	0.012	0.139	0.024	0.016	0.004	0.065
	*A*_B_	0.924	0.080	0.367	1.498	0.685	0.075	0.519	0.811
	*C*	4.29E−4	3.07E−4	−1.75E−6	1.101	—	—	—	—
	*E*	0.271	0.071	0.152	0.439	0.291	0.074	0.167	0.456
	cov*AC*	−0.113	0.005	−0.781	0.115	—	—	—	—
	−2LL	2824.69				2825.45			
	AIC	2846.69				2843.45			
	BIC	2891.87				2880.42			
3. PRS.no.APOE* *+* *ɛ4 alleles	*A*_P_	0.027	0.013	0.025	0.087	0.021	0.015	0.005	0.059
	*A*_e4_	0.118	0.024	0.049	0.242	0.093	0.027	0.046	0.152
	*A*_B_	0.825	0.077	0.318	1.351	0.596	0.075	0.434	0.728
	*C*	4.17E−4	4.94E−4	−5.419E−11	0.474	—	—	—	—
	*E*	0.268	0.069	0.153	0.433	0.289	0.073	0.167	0.451
	cov*AC*	−0.120	0.007	−0.491	0.115	—	—	—	—
	−2LL	3783.69				3784.47			
	AIC	3811.69				3808.47			
	BIC	3869.19				3857.76			

Biometrical analyses of Alzheimer’s disease risk with entry of a PRS were fitted adapting code in Dolan *et al.*^34^ using *OpenMx.*^33^ VC, variance component; Est, Estimate; SE, standard error; LL, lower 95% confidence interval; UL, upper 95% confidence interval; PRS, polygenic risk score at *P *< 1 × 10^−5^ residualized for four PCs and standardized within array type; PRS.no.APOE, PRS without *APOE* region; *A*, additive genetic influences; *C*, common environmental influences; *E*, non-shared environmental influences; *A*_P_, genetic influences due to the PRS; *A*_ɛ4_, genetic influences due to *APOE* ɛ4 alleles; *A*_B_, background additive genetic influences; total *A* = *A*_P_ + *A*_B_ + 2cov*AC*. The Reduced Model dropped *C* (common environmental variance) and cov*AC*. All models adjusted for Sex, LastAge and LastAge^2^. 95% confidence intervals are shown.

**Figure 3:**
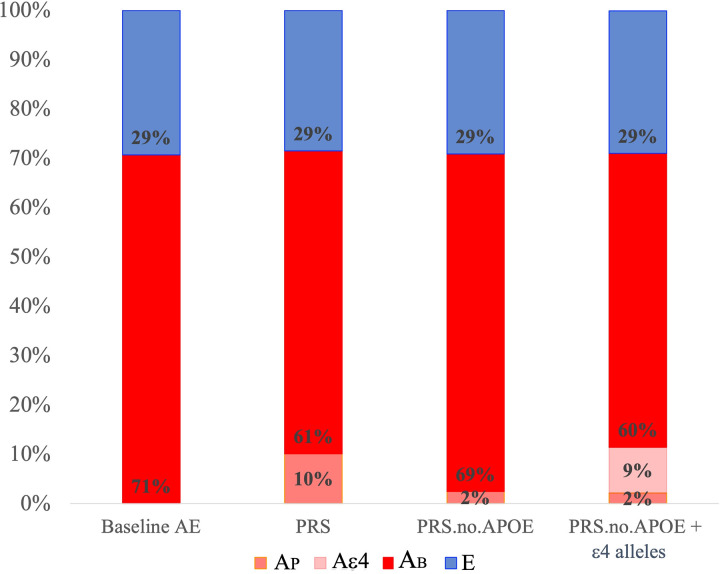
**Biometrical AE model results including Alzheimer’s disease PRSs at the *P *< 1 × 10^−5^ threshold.**  *E*, non-shared environmental influences; *A*, additive genetic influences; *A*_B_, background additive genetic influences; *A*_P_, genetic influences due to a polygenic risk score (PRS); *A*_ɛ4_, genetic influences due to *APOE* ɛ4 alleles. Total *A* = *A*_P_ + *A*_ɛ4_ + *A*_B_ (values from Table 2, Reduced Model). PRSs are based on independent genetic variants reaching a significance threshold of *P *< 1 × 10^−5^ in the GWAS.

Next, we expanded the full baseline ACE model to consider the PRS at *P *< 1 × 10^−5^ as the measured polygenic risk (*A*_P_), remaining background additive genetic (*A*_B_) variance as well as common environmental variance (*C*), the covariance of *A* and *C* (cov*AC*) and *E*. Both *C* and cov*AC* could be dropped (*P* ≥ 5.48 × 10^−1^) (see Supplementary Table 6). In Reduced Model 1, *A*_P_ for the full PRS accounted for 10.1% (σ^2^_*A*__ᴩ_ = 0.101, CI_95_ = 0.051, 0.164) of variation contributing to Alzheimer’s disease risk (see Table 2), whereas, in Reduced Model 2, *A*_P_ for the PRS.no.APOE accounted for 2.4% (σ^2^_*A*ᴩ-*APOE*_ = 0.024, CI_95_ = 0.004, 0.065) (see Table 2). Notably, when *APOE* ɛ4 alleles were added to the model with PRS.no.APOE, the total measured prediction (PRS.no.APOE + ɛ4 alleles) was 11.4% (σ^2^_*A*ᴩ-*APOE *_*= *0.021, CI_95 _= 0.005, 0.059; σ^2^_*A*ɛ4_ = 0.093, CI_95 _= 0.046, 0.152) and the remaining genetic background variance was 59.6% (σ^2^_*A*_ = 0.596, CI_95 _= 0.434, 0.728) (see Fig. 3). Overall, in the context of twin biometrical models, the best measured genetic prediction was observed for directly measured *APOE* ɛ4 alleles_ _+ PRS.no.APOE, but substantial background genetic contributions remain that are not captured by these measured sources.

Our observed power for our given estimate of A of 0.71 was 0.80.^37^ Our observed power for evaluating PRS.no.APOE and *APOE* ɛ4 alleles based on the Reduced Model 3 was 0.77 for PRS.no.APOE and approached 1.00 for *APOE* ɛ4 alleles.

## Discussion

There are many ways to evaluate the importance of genetic influences on Alzheimer’s disease. To date, twin-based models and contributions of PRS have been considered independently. In bringing these approaches together for the first time in the same twin sample, we observed that much of the genetic variance contributing to Alzheimer’s disease liability is not explained by directly measured *APOE* or common genetic influences currently captured by GWAS contributing to a polygenic score. The Alzheimer’s disease PRS contribution to Alzheimer’s disease risk was as high as 0.101, or 10.1% in the twin biometric model. The *APOE* region accounts for much of the measurable contribution to Alzheimer’s disease, with smaller polygenic contribution from other measured common genetic influences. Considering the best biometrical model, directly measured *APOE* ɛ4 explained 9.3% and PRS.no.APOE an additional 2.1% of Alzheimer’s disease risk, leaving much of the genetic risk uncaptured (i.e. 71.1% total minus 11.4% measured).

Our estimates of measured contributions of the PRS to background heritability for Alzheimer’s disease risk, in the same sample, are smaller than the SNP-heritability estimates as well as that for *APOE* ɛ4.^7–9^ While the small contribution of the PRS in this study can potentially be explained by the fact that it is based on the most significant SNPs (*N *= 89),^7^ we note that including PRSs at more relaxed *P*-value thresholds did not pick up more heritability than SNPs with *P *< 1 × 10^−5^. As the GWAS of Alzheimer’s disease is still of comparatively small sample size, based on 21 982 cases and 41 944 controls, this may indicate that substantial genetic variation will be discovered as GWAS sample size increases.

PRS methods rely on the power of GWAS, whereas other genome-wide heritability methods, such as GCTA, are less affected but also often fall short of estimates from twin and family studies.^38^ Moreover, genome-wide methods produce narrow-sense heritability estimates due to additive effects from common SNPs,^8^ whereas twin estimates include both additive and non-additive genetic influences (e.g. dominance and epistasis),^18^ or broad-sense heritability, and with contributions from all variants, common and rare. However, recent work suggests that heritability is ‘recovered’ for complex traits such as human height and body mass index (BMI) when using sequencing data such that SNP-based heritabilities are in line with twin and family-based estimates.^39,40^ Thus, disagreement between biometric and SNP-based heritabilities is not universal. That substantial variation may be attributed to rare variants has also been observed for other complex disease traits such as prostate cancer^41^ and for phenotypes in other species such as yeast.^42,43^ The missing heritability is likely not due to simple additivity across common variants but also to contributions from rare variants as well as to non-additive effects including dominance and epistasis.^42,44^ Studies of rare variants and Alzheimer’s disease risk have observed effects for rare coding variants in genes such as *ABCA7, BIN, NOTCH3, PLCG2, SORL1, TREM* and *ABI3* among others^45–47^ not captured by PRSs. Apart from a rare variant in *TREM2* (p.Arg47His), little replication work has been reported.^8^ However, an Icelandic study observed a protective mutation in the *APP* gene (A673T), that codes for APP, with replication analyses suggesting that it predicted higher cognitive status scores among nursing home residents.^48^

Moreover, gene–environment interplay may increase estimates of genetic influences.^49^ For example, a correlation may be induced between genes and environments (rGE) whereby individuals at higher genetic risk may construct contexts that buffer expression of Alzheimer’s disease, such as engagement in physical or cognitive activities. Empirical examples of rGE for Alzheimer’s disease are rare. On the contrary, studies testing for gene–environment interaction (G × E) are more common for Alzheimer’s disease and related traits, typically evaluating *APOE,*^49–51^ e.g. risk for Alzheimer’s disease is magnified for those with *APOE* risk alleles who are also obese or have high blood pressure in midlife. Moreover, reports from the IGEMS consortium using a within-pair MZ twin design report small-to-moderate G × E effects across country and gender for cross-sectional measures of BMI, depressive symptoms, cognitive performance^52^ as well as grip strength.^53^ Furthermore, *APOE* may partly account for G × E effects for depressive symptoms and spatial reasoning whereby ɛ4 individuals may show less sensitivity to the environment.^52^

In conclusion, in the context of a Swedish twin study, the *APOE* region explains much of the measured genetic contribution to Alzheimer’s disease, with smaller contributions from other measured polygenic influences, yet much of the background genetic liability to risk is unexplained. Sensitive designs that capture all the measured genetic influences, such as the sequencing of rare variants, as well as models that evaluate direct and indirect contributions and gene–environment interplay may reconcile the high background heritability observed in twin and family studies with the extant estimates of measured polygenic risk from genome-wide approaches.

## Supplementary Material

fcab308_Supplementary_Data

## Abbreviations

Aβ =: β-amyloid
APP =: amyloid precursor protein
GWAS =: genome-wide association study
PRS =: polygenic risk score
