# Supplementary material for: Measuring heritable contributions to Alzheimer’s disease: polygenic risk score analysis with twins
Source: Brain Commun. 2022 Jan 4;4(1):fcab308. doi: 10.1093/braincomms/fcab308 (PMC8833403; doi:10.1093/braincomms/fcab308)
Supplement: fcab308_Supplementary_Data [file fcab308_supplementary_data.docx]

**Supplementary Tables 1 - 6**

**Measuring heritable contributions to Alzheimer’s disease: Polygenic risk score analysis with twins**

Ida K. Karlsson, PhD^1,2^, Valentina Escott-Price, PhD ^3^, Margaret Gatz, PhD ^4,1^, John Hardy, PhD^5-9^, Nancy L. Pedersen, PhD^1,10^, Maryam Shoai, PhD^5,6^, Chandra A. Reynolds, PhD^11*^

**Author affiliations:**

1 Department of Medical Epidemiology and Biostatistics, Karolinska Institutet, Stockholm, Sweden

2 Institute of Gerontology and Aging Research Network – Jönköping (ARN-J), School of Health and Welfare, Jönköping University, Jönköping, Sweden

3 UK Dementia Research Institute at Cardiff, Institute of Psychological Medicine and Clinical Neurosciences, Cardiff University, Cardiff, United Kingdom

4 Center for Economic and Social Research, University of Southern California, Los Angeles, CA, USA

5 Department of Neurodegenerative Disease, UCL Queen Square Institute of Neurology, Queen Square, London WC1N 3BG, UK.

6 UK Dementia Research Institute at UCL and Department of Neurodegenerative Disease, UCL Institute of Neurology, University College London, London, UK.

7 Reta Lila Weston Institute, UCL Queen Square Institute of Neurology, 1 Wakefield Street, London WC1N 1PJ, UK

8 UCL Movement Disorders Centre, University College London, London, UK

9 Institute for Advanced Study, The Hong Kong University of Science and Technology, Hong Kong SAR, China

10 Department of Psychology, University of Southern California, Los Angeles, CA, USA

11 Department of Psychology, University of California – Riverside, Riverside, CA, USA

Correspondence to: Chandra A. Reynolds, Department of Psychology, University of California – Riverside, 900 University Ave, 1111 Psychology Bldg, Riverside, CA, USA 92521 chandra.reynolds@ucr.edu

**Supplemental Table 1. Age at last follow-up, dementia onset, or death.**

| AD Status | N | Last Age | SD | Min | Max |
| --- | --- | --- | --- | --- | --- |
| Controls | **1155** | **84.65** | **7.30** | **66.08** | **104.39** |
| SATSA | 458 | 82.09 | 7.26 | 68.08 | 104.39 |
| OCTO-Twin | 33 | 92.60 | 4.82 | 83.08 | 101.95 |
| Gender | 263 | 86.83 | 6.46 | 71.22 | 100.17 |
| Harmony | 401 | 85.49 | 6.92 | 70.07 | 102.09 |
| AD caseS | **431** | **86.97** | **5.87** | **66.05** | **102.26** |
| SATSA | 70 | 87.20 | 6.59 | 66.05 | 97.86 |
| OCTO-Twin | 33 | 92.15 | 4.20 | 85.39 | 102.26 |
| Gender | 63 | 87.81 | 5.00 | 73.88 | 95.50 |
| Harmony | 265 | 86.06 | 5.70 | 71.28 | 100.78 |
| Total | **1586** | **85.28** | **7.02** | **66.05** | **104.39** |

Note. For AD CASES, Last Age = age at dementia diagnosis; For Controls, Last Age = age on December 31, 2016, or age at death, whichever occurred first; for those without register information, age at last study visit was used instead.

**Supplemental Table 2.** Prediction of Alzheimer’s disease risk for AD PRSs at all thresholds.

| Thresholds  Parameters | 5x10^-08^ | 1x10^-05^ | 1x10^-04^ | 1x10^-03^ | .01 | .05 | .50 | 1.00 |
| --- | --- | --- | --- | --- | --- | --- | --- | --- |
| B_PRS_ | 0.37 | 0.38 | 0.38 | 0.36 | 0.28 | 0.19 | 0.22 | 0.21 |
| se | 0.06 | 0.06 | 0.06 | 0.06 | 0.05 | 0.05 | 0.05 | 0.05 |
| *P* | 1.64E-11 | 7.53E-12 | 6.50E-12 | 2.55E-10 | 1.63E-07 | 1.36E-04 | 1.97E-05 | 4.10E+00 |
| AUC | 0.972 | 0.972 | 0.972 | 0.974 | 0.975 | 0.976 | 0.976 | 0.976 |
| R^2^ Nagelkerke | 0.058 | 0.062 | 0.062 | 0.053 | 0.033 | 0.016 | 0.021 | 0.019 |
|  |  |  |  |  |  |  |  |  |
| Thresholds  Parameters | **5x10^-08^** | **1x10^-05^** | **1x10^-04^** | **1x10^-03^** | **.01** | **.05** | **.50** | **1.00** |
| B_PRS.No.APOE_ | 0.16 | 0.16 | 0.17 | 0.17 | 0.16 | 0.11 | 0.16 | 0.16 |
| se | 0.05 | 0.05 | 0.05 | 0.05 | 0.05 | 0.05 | 0.05 | 0.05 |
| *P* | 1.39E-03 | 1.41E-03 | 7.55E-04 | 3.48E-04 | 1.18E-03 | 2.05E-02 | 9.46E-04 | 1.74E-03 |
| AUC | 0.977 | 0.977 | 0.976 | 0.976 | 0.977 | 0.976 | 0.976 | 0.976 |
| R^2^ Nagelkerke | 0.011 | 0.011 | 0.012 | 0.013 | 0.011 | 0.006 | 0.012 | 0.011 |

*Note*. Analyses adjusted for Twin type (MZ or DZ), Sex (0=M, 1=F), Last Age (age at last follow-up, death, or dementia onset, centered on age 80 years and divided by 10), Array type (Human OmniExpress = 0, Illumina PsychArray =1). PRSs were residualized for 4 PCs and standardized within array type. Random effects estimated for MZ and DZ pairs. AUC=Area under the curve.

**Supplemental Table 3**. Logistic Regression analyses (*N* = 1586): AD PRS at *P*<1x10^-05^.

|  | **Baseline** | | | **PRS *P*<1x10^-05^** | | | **PRS.no.APOE *P*<1x10^-05^** | | | **PRS.no.APOE + *APOE* alleles** | | |
| --- | --- | --- | --- | --- | --- | --- | --- | --- | --- | --- | --- | --- |
| **Parameters** | **B** | **se** | ***P*(>\|z\|)** | **B** | **se** | ***P*(>\|z\|)** | **B** | **se** | ***P*(>\|z\|)** | **B** | **se** | ***P*(>\|z\|)** |
| Intercept | -2.68 | 0.36 | 5.49E-14 | -2.90 | 0.38 | 1.13E-14 | -2.72 | 0.36 | 3.64E-14 | -3.16 | 0.39 | 4.44E-16 |
| MZ | 0.04 | 0.29 | 8.80E-01 | 0.10 | 0.26 | 7.08E-01 | 0.11 | 0.28 | 6.84E-01 | 0.12 | 0.26 | 6.26E-01 |
| Sex (0=M, 1=F) | 0.76 | 0.16 | 4.21E-06 | 0.77 | 0.17 | 3.80E-06 | 0.77 | 0.17 | 3.36E-06 | 0.73 | 0.17 | 1. 20E-05 |
| Last Age | 1.74 | 0.23 | 5.88E-14 | 1.74 | 0.24 | 1.92E-13 | 1.71 | 0.23 | 2.29E-13 | 1.72 | 0.24 | 3.30E-13 |
| Last Age^2^ | -1.00 | 0.16 | 5.31E-10 | -0.90 | 0.16 | 1.40E-08 | -0.97 | 0.16 | 1.79E-09 | -0.86 | 0.16 | 4.13E-08 |
| Array | 0.69 | 0.29 | 1.67E-02 | 0.77 | 0.30 | 1.07E-02 | 0.68 | 0.29 | 1.78E-02 | 0.65 | 0.30 | 3.17E-02 |
| PRS | -- | -- | -- | 0.67 | 0.10 | 1.79E-11 | 0.27 | 0.09 | 1.53E-03 | 0.29 | 0.09 | 1.05E-03 |
| APOE ε2 alleles | -- | -- | -- | -- | -- | -- | -- | -- | -- | -0.31 | 0.22 | 1.52E-01 |
| APOE ε4 alleles | -- | -- | -- | -- | -- | -- | -- | -- | -- | 1.29 | 0.19 | 5.18E-12 |
| Random.MZ | 6.88 | 2.39 | 4.08E-03 | 5.34 | 1.91 | 5.31E-03 | 6.31 | 2.27 | 5.40E-03 | 4.81 | 1.77 | 6.46E-03 |
| Random.DZ | 1.03 | 0.66 | 1.18E-01 | 1.16 | 0.72 | 1.08E-01 | 1.11 | 0.69 | 1.10E-01 | 1.03 | 0.71 | 1.46E-01 |
| Fit Statistics |  |  |  |  |  |  |  |  |  |  |  |  |
| Deviance | 1702.30 |  |  | 1636.80 |  |  | 1691.07 |  |  | 1609.54 |  |  |
| AIC | -859.15 |  |  | -827.40 |  |  | -854.54 |  |  | -815.77 |  |  |
| SBC | -879.30 |  |  | -850.06 |  |  | -877.20 |  |  | -843.47 |  |  |
| ICC.MZ | 0.676 |  |  | 0.619 |  |  | 0.657 |  |  | 0.594 |  |  |
| ICC.DZ | 0.238 |  |  | 0.260 |  |  | 0.252 |  |  | 0.239 |  |  |
| AUC | 0.929 |  |  | 0.912 |  |  | 0.925 |  |  | 0.906 |  |  |
| R^2^ Nagelkerke | 0.085 |  |  | 0.061 |  |  | 0.011 |  |  | 0.076 |  |  |

*Note*. MZ= Monozygotic twin; DZ= dizygotic twin; Last Age = age at last follow-up, death, or dementia onset, centered on age 80 years and divided by 10; Array = Human OmniExpress = 0, Illumina PsychArray =1; PRS = PRS at *p*<1x10^-05^ residualized for 4 PCs and standardized within array type; PRS.no.APOE = PRS without *APOE* region; Random = random effect; Deviance = -2*ln*(Likelihood); AIC=Akaike Information Criteria; SBC= Schwarz Bayesian criterion; ICC = intraclass correlation measured as Random.MZ / (3.29+ Random.MZ) and Random.DZ / (3.29+ Random.DZ ).^1^ AUC=Area under the curve.

**Supplemental Table 4**. Probit regression analyses in complete pairs (*N* = 898, N_pairs_= 449): AD PRS at *P*<1x10^-05^.

|  | **Baseline** | | | **PRS *P*<1x10^-05^** | | | **PRS.no.APOE *P*<1x10^-05^** | | | **PRS.no.APOE + *APOE* alleles** | | |
| --- | --- | --- | --- | --- | --- | --- | --- | --- | --- | --- | --- | --- |
| **Parameters** | **B** | **se** | ***P*(>\|z\|)** | **B** | **se** | ***P*(>\|z\|)** | **B** | **se** | ***P*(>\|z\|)** | **B** | **se** | ***P*(>\|z\|)** |
| Intercept | -1.36 | 0.24 | 1.35E-08 | -1.47 | 0.25 | 4.95E-09 | -1.34 | 0.24 | 1.32E-08 | -1.54 | 0.26 | 3.28E-09 |
| MZ | 0.16 | 0.22 | 4.74E-01 | 0.14 | 0.21 | 4.94E-01 | 0.19 | 0.21 | 3.83E-01 | 0.16 | 0.20 | 4.30E-01 |
| Sex (0=M, 1=F) | 0.31 | 0.13 | 1.92E-02 | 0.31 | 0.13 | 2.08E-02 | 0.30 | 0.13 | 2.44E-02 | 0.30 | 0.13 | 2.50E-02 |
| Last Age | 0.92 | 0.15 | 8.99E-10 | 0.93 | 0.16 | 4.24E-09 | 0.89 | 0.15 | 4.11E-09 | 0.92 | 0.16 | 3.99E-09 |
| Last Age^2^ | -0.54 | 0.12 | 1.35E-05 | -0.50 | 0.13 | 6.59E-05 | -0.52 | 0.12 | 2.52E-05 | -0.47 | 0.13 | 1.72E-04 |
| Array | 0.04 | 0.21 | 8.64E-01 | 0.09 | 0.22 | 6.88E-01 | 0.00 | 0.21 | 9.89E-01 | -0.03 | 0.23 | 8.85E-01 |
| PRS | -- | -- | -- | 0.44 | 0.08 | 8.92E-09 | 0.21 | 0.07 | 3.30E-03 | 0.21 | 0.07 | 2.98E-03 |
| APOE ε2 alleles | -- | -- | -- | -- | -- | -- | -- | -- | -- | -0.39 | 0.20 | 5.05E-02 |
| APOE ε4 alleles | -- | -- | -- | -- | -- | -- | -- | -- | -- | 0.77 | 0.15 | 3.81E-07 |
| Random.MZ | 2.48 | 0.87 | 4.39E-03 | 2.24 | 0.81 | 5.48E-03 | 2.38 | 0.87 | 6.09E-03 | 2.02 | 0.76 | 7.53E-03 |
| Random.DZ | 0.28 | 0.21 | 1.82E-01 | 0.24 | 0.21 | 2.65E-01 | 0.28 | 0.22 | 2.00E-01 | 0.21 | 0.22 | 3.30E-01 |
| Fit Statistics |  |  |  |  |  |  |  |  |  |  |  |  |
| Deviance | 873.58 |  |  | 832.06 |  |  | 864.18 |  |  | 820.06 |  |  |
| AIC | -444.79 |  |  | -425.03 |  |  | -441.09 |  |  | -421.03 |  |  |
| SBC | -461.22 |  |  | -443.51 |  |  | -459.57 |  |  | -443.62 |  |  |
| ICC.MZ | 0.713 |  |  | 0.691 |  |  | 0.704 |  |  | 0.669 |  |  |
| ICC.DZ | 0.219 |  |  | 0.188 |  |  | 0.218 |  |  | 0.173 |  |  |
| AUC | 0.960 |  |  | 0.958 |  |  | 0.962 |  |  | 0.955 |  |  |
| R^2^ Nagelkerke | 0.067 |  |  | 0.073 |  |  | 0.017 |  |  | 0.078 |  |  |

*Note*. MZ= Monozygotic twin; DZ= dizygotic twin; Last Age = age at last follow-up, death, or dementia onset, centered on age 80 years and divided by 10; Array = Human OmniExpress = 0, Illumina PsychArray =1; PRS = PRS at *p*<1x10^-05^ residualized for 4 PCs and standardized within array type; PRS.no.APOE = PRS without *APOE* region; Random = random effect; Deviance = -2*ln*(Likelihood); AIC=Akaike Information Criteria; SBC= Schwarz Bayesian criterion; ICC = intraclass correlation measured as Random.MZ / (1+ Random.MZ) and Random.DZ / (1+ Random.DZ).^2^ AUC=Area under the curve.

**Supplemental Table 5. Probit regression analyses (*N* = 1586): Alzheimer’s disease PRS at *P*<1x10^-05^: adjusted for sub-study**

|  | **Baseline** | | | **PRS *P*<1x10^-05^** | | | **PRS.no.APOE *P*<1x10^-05^** | | | **PRS.no.APOE + *APOE* alleles** | | |
| --- | --- | --- | --- | --- | --- | --- | --- | --- | --- | --- | --- | --- |
| **Parameters** | **B** | **se** | ***P*(>\|z\|)** | **B** | **se** | ***P*(>\|z\|)** | **B** | **se** | ***P*(>\|z\|)** | **B** | **se** | ***P*(>\|z\|)** |
| Intercept | -0.93 | 0.18 | 2.07E-07 | -1.04 | 0.19 | 2.85E-08 | -0.94 | 0.18 | 1.61E-07 | -1.20 | 0.19 | 5.83E-10 |
| MZ | 0.04 | 0.14 | 7.53E-01 | 0.03 | 0.13 | 8.02E-01 | 0.07 | 0.14 | 5.94E-01 | 0.06 | 0.13 | 6.52E-01 |
| Harmony [ref] | [0] |  |  |  |  |  |  |  |  |  |  |  |
| SATSA | -0.97 | 0.13 | 1.62E-13 | -0.89 | 0.13 | 4.56E-12 | -0.95 | 0.13 | 4.59E-13 | -0.89 | 0.13 | 6.16E-12 |
| Octo-Twin | 0.19 | 0.22 | 4.00E-01 | 0.27 | 0.21 | 2.00E-01 | 0.18 | 0.22 | 4.09E-01 | 0.23 | 0.21 | 2.72E-01 |
| GENDER | -0.61 | 0.12 | 2.87E-07 | -0.61 | 0.12 | 7.39E-07 | -0.63 | 0.12 | 2.52E-07 | -0.57 | 0.12 | 2.78E-06 |
| Sex (0=M, 1=F) | 0.48 | 0.09 | 1.62E-07 | 0.47 | 0.09 | 4.65E-07 | 0.48 | 0.09 | 1.51E-07 | 0.44 | 0.09 | 1.59E-06 |
| Last Age | 0.77 | 0.12 | 1.80E-10 | 0.78 | 0.12 | 2.80E-10 | 0.76 | 0.12 | 5.18E-10 | 0.77 | 0.12 | 5.23E-10 |
| Last Age^2^ | -0.48 | 0.08 | 1.18E-08 | -0.44 | 0.08 | 1.76E-07 | -0.47 | 0.08 | 4.05E-08 | -0.42 | 0.08 | 5.23E-07 |
| Array | 0.23 | 0.15 | 1.18E-01 | 0.28 | 0.16 | 7.61E-02 | 0.23 | 0.15 | 1.26E-01 | 0.22 | 0.16 | 1.56E-01 |
| PRS | -- | -- | -- | 0.33 | 0.05 | 1.78E-11 | 0.14 | 0.05 | 3.24E-03 | 0.14 | 0.05 | 2.39E-03 |
| *APOE* ε2 alleles | -- | -- | -- | -- | -- | -- | -- | -- | -- | -0.13 | 0.12 | 2.53E-01 |
| *APOE* ε4 alleles | -- | -- | -- | -- | -- | -- | -- | -- | -- | 0.66 | 0.09 | 3.04E-12 |
| Random.MZ | 1.39 | 0.49 | 4.88E-03 | 1.12 | 0.44 | 1.12E-02 | 1.24 | 0.47 | 8.10E-03 | 0.99 | 0.42 | 1.76E-02 |
| Random.DZ | 0.17 | 0.17 | 3.25E-01 | 0.18 | 0.18 | 3.33E-01 | 0.18 | 0.18 | 3.11E-01 | 0.16 | 0.19 | 3.87E-01 |
| **Fit Statistics** |  |  |  |  |  |  |  |  |  |  |  |  |
| Deviance | 1613.77 |  |  | 1557.32 |  |  | 1604.27 |  |  | 1532.54 |  |  |
| AIC | -817.89 |  |  | -790.66 |  |  | -814.13 |  |  | -780.27 |  |  |
| SBC | -845.59 |  |  | -820.88 |  |  | -844.35 |  |  | -815.52 |  |  |
| ICC.MZ | 0.582 |  |  | .528 |  |  | 0.554 |  |  | 0.498 |  |  |
| ICC.DZ | 0.145 |  |  | .150 |  |  | 0.154 |  |  | 0.139 |  |  |
| AUC | 0.954 |  |  | 0.953 |  |  | 0.955 |  |  | 0.950 |  |  |
| R^2^ Nagelkerke | 0.160 |  |  | 0.055 |  |  | 0.009 |  |  | 0.069 |  |  |

*Note*. Regressions analyses estimating varying intraclass correlations (ICCs) with clustered twin data were adapted from code in Archer et al (2015) using *mixor.*^2^ MZ= Monozygotic twin; DZ= dizygotic twin; Last Age = age at last follow-up, death, or dementia onset, centered on age 80 years and divided by 10; Array = Human OmniExpress = 0, Illumina PsychArray =1; PRS = PRS at *p*<1x10^-05^ residualized for 4 PCs and standardized within array type; PRS.no.APOE = PRS without *APOE* region; Random = random effect; Deviance = -2*ln*(Likelihood); AIC=Akaike Information Criteria; SBC= Schwarz Bayesian criterion; ICCs measured as Random.MZ / (1+ Random.MZ) and Random.DZ / (1+ Random.DZ);^2^ AUC=Area under the curve.

**Supplemental Table 6.** Biometrical model fit comparisons.

| **Model** | | **base** | **comparison** | **ep** | **-2LL** | **df** | **AIC** | $\boldsymbol{\Delta}$**-2LL** | $\boldsymbol{\Delta}$**df** | ***P*** |
| --- | --- | --- | --- | --- | --- | --- | --- | --- | --- | --- |
| **0** | Baseline | ACE | -- | 7 | 888.71 | 893 | 902.71 | -- | -- | -- |
|  |  | ACE | AE | 6 | 889.52 | 894 | 901.52 | 0.81 | 1 | 3.69E-01 |
|  |  | ACE | CE | 6 | 899.84 | 894 | 911.84 | 11.13 | 1 | 8.51E-04 |
|  |  | ACE | E | 5 | 939.57 | 895 | 949.57 | 50.86 | 2 | 9.03E-12 |
| **1** | PRS | A_P_A_B_CE, covAC | -- | 11 | 2746.32 | 1596 | 2768.32 | -- | -- | -- |
|  |  | A_P_A_B_CE, covAC | A_P_A_B_CE | 10 | 2747.52 | 1597 | 2767.52 | 1.20 | 1 | 2.73E-01 |
|  |  | A_P_A_B_CE, covAC | A_P_A_B_E | 9 | 2747.52 | 1598 | 2765.52 | 1.20 | 2 | 5.48E-01 |
| **2** | PRS.no.APOE | A_P_A_B_CE, covAC | -- | 11 | 2824.69 | 1596 | 2846.69 | -- | -- | -- |
|  |  | A_P_A_B_CE, covAC | A_P_A_B_CE | 10 | 2825.45 | 1597 | 2845.45 | 0.76 | 1 | 3.82E-01 |
|  |  | A_P_A_B_CE, covAC | A_P_A_B_E | 9 | 2825.45 | 1598 | 2843.45 | 0.76 | 2 | 6.82E-01 |
| **3** | PRS.no.APOE | A_P_A_e4_A_B_CE, covAC | -- | 14 | 3783.69 | 2301 | 3811.69 | -- | -- | -- |
|  | + $\varepsilon$4 alleles | A_P_A_e4_A_B_CE, covAC | A_P_A_ε4_A_B_CE | 13 | 3784.47 | 2302 | 3810.47 | 0.78 | 1 | 3.77E-01 |
|  |  | A_P_A_e4_A_B_CE, covAC | A_P_A_ε4_A_B_E | 12 | 3784.47 | 2303 | 3808.47 | 0.78 | 2 | 6.77E-01 |

***Note***. ep = estimated parameters; LL = Log Likelihood; AIC = Akaike Information Criteria; df = degrees of freedom; PRS = PRS at *p*<1x10^-05^ residualized for 4 PCs and standardized within array type; PRS.no.APOE = PRS without *APOE* region. A = total additive genetic influences; A_P_ = genetic influences due to the polygenic score (PRS), A _ε4_ = genetic influences due to *APOE* ε4 alleles, and A_B_ = background additive genetic influences; C = common environmental influences that are perfectly correlated among both MZ and DZ pairs; E = non-shared environmental influences. Adjusted for Sex, LastAge and LastAge^2^.

**References**

1. Wu S, Crespi CM, Wong WK. Comparison of methods for estimating the intraclass correlation coefficient for binary responses in cancer prevention cluster randomized trials. *Contemp Clin Trials*. Sep 2012;33(5):869-80. doi:10.1016/j.cct.2012.05.004

2. Archer KJ, Hedeker D, Nordgren R, Gibbons RD. mixor: An R Package for Longitudinal and Clustered Ordinal Response Modeling. 2015;
